# An incidentally found mass on the remnant stomach after a Roux-en-Y gastric bypass

**DOI:** 10.1186/s40792-024-01953-3

**Published:** 2024-06-20

**Authors:** Jennifer E. Geller, Santosh Swaminathan, Kristin Noonan

**Affiliations:** 1 Department of Surgery, Thomas Jefferson University Hospital, 1025 Walnut St, Philadelphia, PA 19107 USA; 2 Department of Surgery, Jefferson Abington Hospital, Abington, PA USA

**Keywords:** Gastrointestinal stromal tumors, Incidental findings, Surgical resection, Bariatric surgery

## Abstract

**Background:**

Incidentally found masses are a widely discussed area of medicine, and there are conflicting opinions as to how to deal with these findings, particularly in the stomach—which has limited documentation in the literature. Here we present a middle-aged female who was found to have an incidentally found mass on her remnant stomach 10 years after a Roux-en-Y gastric bypass (RYGB) surgery.

**Case presentation:**

We present the case of a 66-year-old female who is 10 years post-op from a RYGB. After a bout of self-resolving diarrheal illness prompted a computed tomography (CT) scan in the emergency department, she was diagnosed with a 9-cm mass on her remnant stomach that after resection was found to be a gastrointestinal stromal tumor (GIST) with the PDGRRA p.D842V gene mutation.

**Conclusion:**

The National Comprehensive Cancer Network (NCCN) outlines guidelines for the workup of abdominal masses. While endoscopic ultrasound is a common step in diagnosis of gastric masses, for a patient who has had a RYGB, access to the remnant stomach, which is no longer a part of the alimentary tract, is not possible. Thus, this patient’s mass was surgically resected. Given the low risk of recurrence, her future care consists of follow-up with medical oncology in accordance with the NCCN guidelines.

## Introduction

**Fig. 1:**
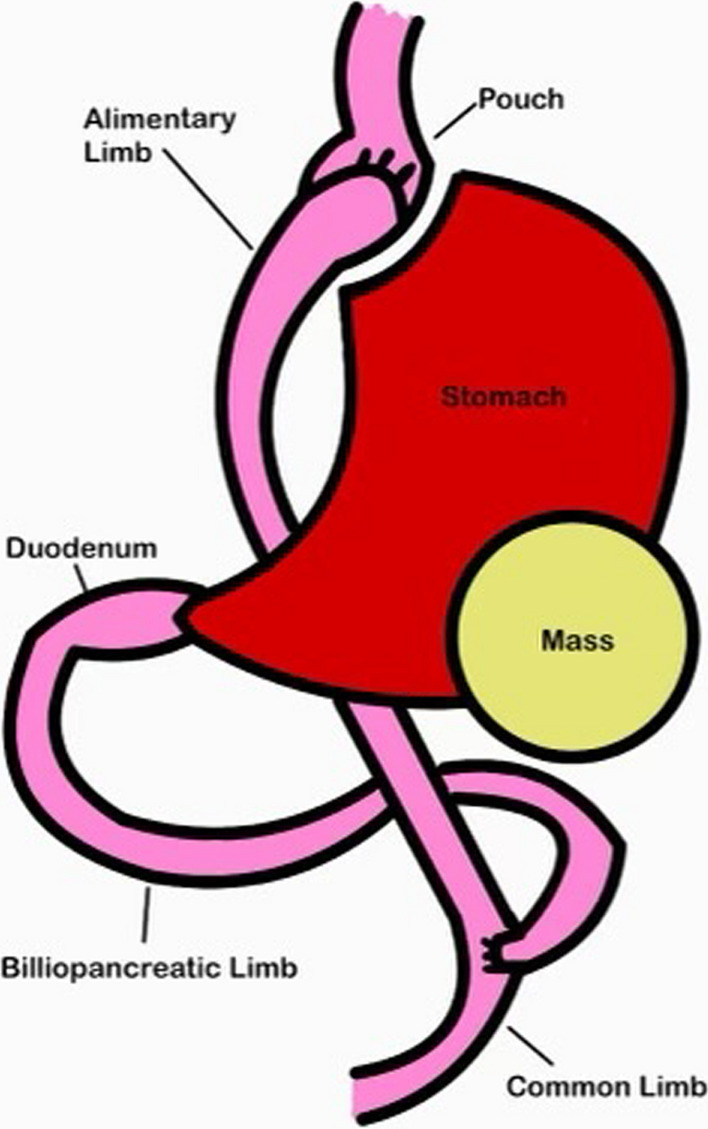
Graphic depicting RYGB

The more frequent use of imaging modalities such as computed tomography (CT) scans has increased incidental findings on imaging [1]. Depending on the pathology of the finding, surgical management may be warranted whereas in other pathologies, medical management is sufficient [2, 3]. Roux-en-Y gastric bypass (RYGB) is a primary bariatric surgical procedure aimed at helping patients achieve weight loss. The creation of the bypass involves creation of a gastric pouch that is connected to the alimentary tract via gastrojejunostomy. A biliopancreatic limb that is connected to a now not function part of the stomach is reconnected with the alimentary tract at a created jejunojejunostomy (Fig. 1) [4]. The not functional part of the stomach that is excluded in this process is left in place and is called the remnant stomach.

We present a case of a middle-aged woman who presented to the emergency department for self-resolving gastrointestinal illness, but whose workup led to the discovery of an incidentally found mass on the patient’s remnant stomach.

## Case presentation

A 66-year-old female with a past history of gastroesophageal reflux disease, appendectomy, and RYGB 10 years ago, presented to the emergency department (ED) due to epigastric pain and nausea. When in the ED, the workup for her abdominal pain included bloodwork and an abdominal CT scan.

**Fig. 2:**
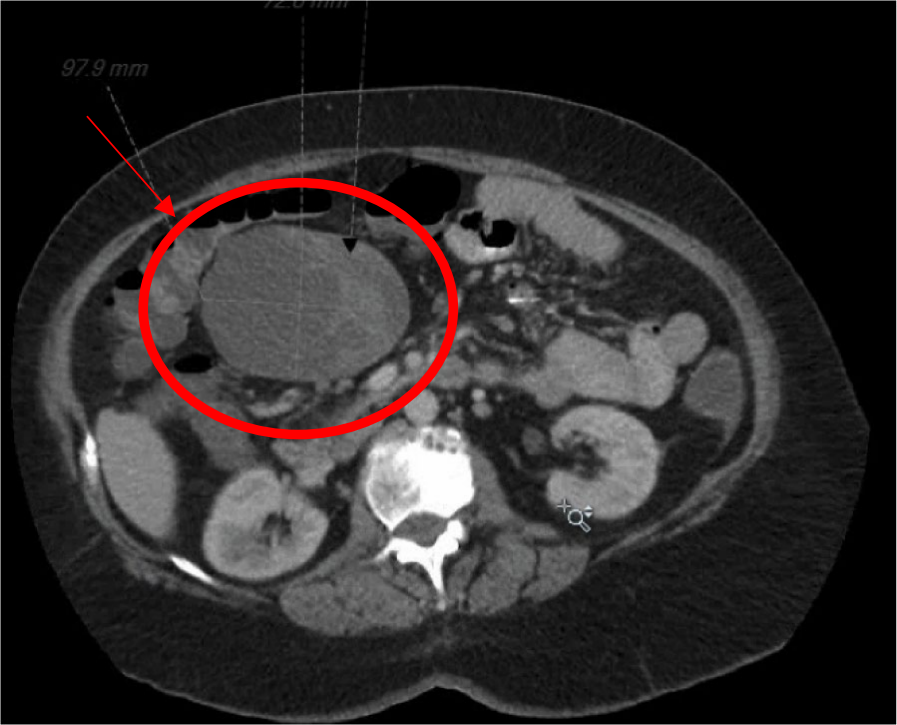
Incidentally found abdominal mass arising from the patient’s remnant stomach

The patient appeared in no acute distress, and her abdominal exam was benign with a soft, nontender, non-distended abdomen. She was afebrile, normotensive, and her heart rate was within normal limits. Her labs were all within normal limits including her white blood cell count of 9.6 k WBC/L and normal liver function tests. However, a CT scan of her abdomen and pelvis revealed a 9.8 × 7.2 × 8.4 cm complex heterogenous cystic lesion arising from the inferior aspect of the gastric antrum that demonstrated thick enhancing septae (Fig. 2).

The patient’s symptoms subsequently resolved after a bowel movement in the emergency department—and thus were deemed unrelated to the mass and more likely gastroenteritis. However, given the possible challenge of endoscopic ultrasound given the patient’s anatomy in the setting of her RYGB, after discussion with the patient, she decided to undergo surgical resection of her remnant stomach and the mass by the bariatric surgery team.

**Fig. 3:**
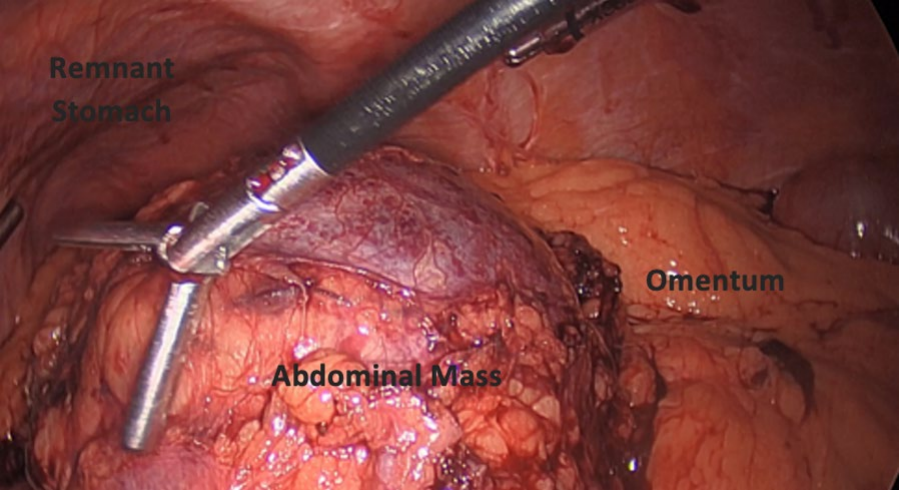
Laparoscopic imaging of the gastric remnant and mass

The patient returned for the operation 2 months later. Upon preparation for laparoscopy via trocar placement and liver retraction, the large cystic mass was visualized under the greater omentum (Fig. 3). The mass was resected by working from the top of the remnant stomach downward. The roux limb was mobilized off of the remnant first before the remnant was resected off of the spleen and left crus. The remnant was separated from the pouch, and the lesser and greater curve were released with harmonic while transecting the duodenal bulb. The Roux limb was sutured to the retroperitoneum to close the space behind the Roux limb.

**Fig. 4:**
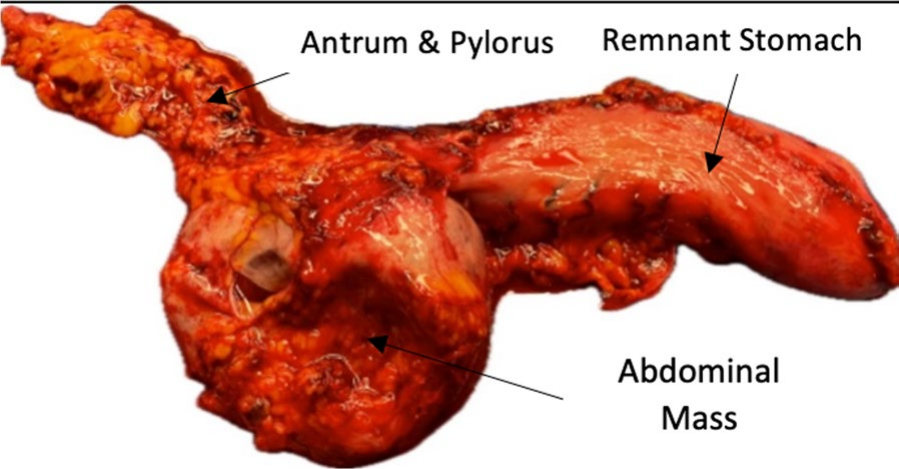
Resected gastric mass attached to the remnant stomach

The remnant and mass were removed from the abdomen en bloc to prevent peritoneal seeding. The specimen was brought to a side table for opening and inspection (Fig. 4). The patient’s postoperative course was uncomplicated; she was discharged on postoperative day 1 and proceeded to have an uncomplicated recovery.

The pathology results reported a diagnosis of a gastrointestinal stromal tumor (GIST). The mass was a 9-cm-long pedunculated heterogenous mass that was 2/3 solid and 1/3 cystic. The mitotic rate was noted to be 1 mitotic event for every 5 mm^2^, and there were negative margins and benign lymph nodes. Further genetic testing of the mass indicated a PDGRRA p.D842V mutation oncogenic exon 18 mutation. Given her low risk of recurrence, she did not go on to receive adjuvant chemotherapy, and continued with surveillance with medical oncology.

## Discussion

This case report is unique in that it highlights how deviations from standard anatomy require careful planning for operative techniques in management and treatment of abdominal masses. Only one case report was located regarding this issue which was regarding an exploratory laparotomy with distal pancreatectomy, partial colectomy, partial gastrectomy, and splenectomy given infiltration of that mass toward the celiac vessels and surrounding organs [5]. Endoscopic ultrasound (EUS) is a commonly used tool in the diagnosis and staging of gastric tumors in order to assess gastric wall involvement and if there is a presence of infiltration in the paragastric lymph nodes [6]. However, there are cases when this is not done or is not possible, and when a patient has a RYGB, the endoscopic route is complicated by the separated remnant stomach such that a mass at the distal end of the remnant would not be accessible on endoscopy. Typically, per the National Comprehensive Cancer Network (NCCN) guidelines for workup of GIST, for a very small gastric GIST (under 2 cm^2^), EUS in conjunction with CT or MRI of the patient’s abdomen and pelvis can be used, which after that time if the tumor is found to have irregular borders, cystic spaces, ulceration, echogenic foci, and heterogeneity, surgical resection would be warranted [7]. However, per the NCCN, if the mass is known to be suspicious for GIST clinically or there is a known mass based on previous imaging, a chest X-ray imaging becomes warranted, and the surgeon may proceed to resection. Positron emission tomography can be used, but per NCCN does not replace diagnostic CT imaging.

GISTs are abnormal cells of the interstitial cells of Cajal, which are mesenchymal cells known to help move food along in the GI tract [8]. Most commonly found in the stomach, 80% of GISTs have the c-Kit mutation and 10% have the PDGFRA gene, all typically with the CD117 marker. GISTs are often asymptomatic. The decision for adjuvant chemotherapy is based on a number of factors. Of note, patients with c-kit mutations are typically given imatinib as the drug of choice, however, the PDGFRA mutation is resistant to imatinib [9]. Secondly, adjuvant chemotherapy is reserved for those with high-risk features. Per NCCN, risk stratification is based on mitotic rate, location, and size [7]. Gastric tumors smaller than 10 cm, with a mitotic rate under 10 per 50 HPF that have no peritoneal seeding are low risk and do not need adjuvant chemotherapy. Thus, our patient was considered low risk and adjuvant chemotherapy was not indicated.

Our patient followed up with medical oncology in accordance with NCCN guidelines for low-risk disease. This includes follow-up appointments with an oncology with repeat CT imaging of her abdomen and pelvis—MRI is also acceptable—every 3 to 6 months for 5 years then annually if there is no recurrence. If a patient were to be high-risk, 3-month follow-up visits would be indicated [7].

## Conclusion

For patients who have GISTs and other gastric abnormalities, endoscopic ultrasound is the primary diagnostic modality. However, in patients who have undergone RYGB, access to the remnant stomach, which is no longer a part of the alimentary tract, is more challenging and would require surgical assistance. Thus, surgical resection of the mass directly in this patient allowed for diagnosis and treatment of the GIST. With a low risk of recurrence based on tumor features, follow-up with medical oncology is sufficient and she does not require adjuvant chemotherapy.

## Data Availability

Data not available - participant consent.
